# Editorial: Coronavirus Research Landscape: Resources, Utilities, and Analytic Studies

**DOI:** 10.3389/frma.2021.712672

**Published:** 2021-06-25

**Authors:** Chaomei Chen, David Chavalarias, Neil R. Smalheiser, Dietmar Wolfram

**Affiliations:** ^1^ College of Computing and Informatics, Drexel University, Philadelphia, PA, United States; ^2^ Centre d’Analyse et de Mathématique Sociales, École des Hautes Études en Sciences Sociales, Paris, France; ^3^ Department of Psychiatry, University of Illinois at Chicago, Chicago, llinois, United States; ^4^ School of Information Studies, University of Wisconsin-Milwaukee, Milwaukee, WI, United States

**Keywords:** COVID-19, coronavirus research, resources of scholarly publications, studies of scholarly literature, literature reviews

**Editorial on the Research Topic**

**Coronavirus Research Landscape: Resources, Utilities, and Analytic Studies**

## Overview

The full impact of the COVID-19 pandemic has yet to be realized. It is not just a health issue but one that has affected how the society functions. Since the discovery of the coronavirus responsible for COVID-19, an explosion of research has been published on different aspects of the disease. The resulting data and literature emerging from these efforts warrant investigation to better understand how the COVID-19 research landscape has evolved, particularly in view of its relatively short time span. This Research Topic features five research articles and one opinion article on various aspects of the COVID-19 research landscape.

The COVID-19 Research Dataset (CORD-19) is one of the high-profile datasets of the research literature of COVID-19. Kanakia et al. outline a framework for examining biases in datasets such as CORD-19. They demonstrate how three expansions of CORD-19 may better capture the breadth of the relevant research and reduce the topical coverage biases of the original CORD-19.

Hook et al. demonstrate how another large-scale resource of research literature can be used to analyze COVID-19 research, namely the Dimensions platform from Digital Science. The Dimensions database consists of a large collection of research inputs, outputs and the attention accrued by research objects of interest. The authors introduce the idea of “real-time” bibliometrics using the Dimensions data that allow for more timely access to, and analysis of, scholarly data. The authors present analyses of scholarly communication patterns during this rapidly developing research front, including collaboration patterns and gender imbalances evident in the literature.

The Research Topic also includes three analytic studies of the COVID-19 research. Porter et al. delineate a profile of COVID-19-related research indexed in the PubMed database so as to make research findings more accessible to the research community and to link research findings to COVID-19 concerns through Literature-Based Discovery (LBD) techniques. The analysis addresses Who, When, Where and What issues associated with the literature and research landscape. The authors also analyze emerging topics based on terms appearing in titles and abstracts. The methods used serve as examples of how LBD techniques may be applied in the development of research profiles.

Mei et al. investigate the intellectual structure of coronavirus research using author co-citation analysis based on data from Clarivate Analytics’ Web of Science Core Collection and the PubMed database. Author co-citation methods represent a longstanding approach to better understand collaboration patterns and research areas. Using factor analysis and associated visualizations, the authors identified four primary dimensions: outbreaks, viral structure and function, vaccine and therapeutic development, and coronavirus in various animals. They also noted less prominent themes in the existing research such as mental health issues and the social and economic disruption caused by the pandemic.

Chen introduces a generic and reproducible method for data collection and sense-making of the rapidly developing COVID-19 research environment based on Microsoft Academic Graph (MAG). The citation contexts and associated uncertainties of the first 8 months of COVID-19 literature are analyzed to illustrate the potential of the visual analytic method using CiteSpace, a tool developed by the author for conducting visual analytic studies of scholarly literature. The author outlines the benefits of the developed method and demonstrates how it may be applied to other research areas.

Last, but not least, in their opinion article, Orellana-Serradell et al. examine the broader issue of scholarly peer review in the context of COVID-19. The authors analyze the content of selected COVID-19 papers published in prestigious journals and compare them with non-COVID-19 publications. They show that the COVID-19 papers were more likely to be missing details of the studies and how they were carried out, in comparison to non-COVID-related control papers.

We hope this research topic collectively leads to a better understanding of the research landscape concerning the pandemic and beyond. We are grateful to the reviewers of the research topic for their valuable time and constructive efforts.

